# Supplementary material for: Population-level control of two manganese oxidases expands the niche for bacterial manganese biomineralization
Source: NPJ Biofilms Microbiomes. 2025 Mar 24;11:50. doi: 10.1038/s41522-025-00670-5 (PMC11930936; doi:10.1038/s41522-025-00670-5)
Supplement: Supplementary file 1 — Supplementary Information [file 41522_2025_670_MOESM1_ESM.pdf]

Supplementary Information for

Population-level control of two manganese oxidases expands the niche for bacterial manganese biomineralization

Gaitan Gehin[1], Nicolas Carraro[2], Jan Roelof van der Meer[2], Jasquelin Peña[1,3]

[1]Department of Civil and Environmental Engineering, University of California, Davis, United States

[2]Department of Fundamental Microbiology, University of Lausanne, Switzerland

[3]Energy Geosciences Division, Lawrence Berkeley National Laboratory

Jasquelin Peña

Email: pena@ucdavis.edu

This PDF file includes:

Supplementary Figures 1 to 22

Supplementary Tables S1 to S4

mnxG (2447) and promoter area (P2447)

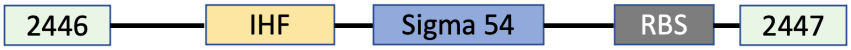

TGGGGCTTTGACGGCCACTTTCCATACTGGACTGGGTTGCCTGATGGGCCCGTCGATCTTGTACTTTTGTGTTCGCGGTCTGGTCGG
C**TGA**CAGCGAATTCGTGGGAGCCGCCGCATCGACATCAAACGCTGCGATGCGGCACCCCCCGATTCTGGGAAATCCGTTGGGGGATC
CTCCCCAATCTGTTTCGGTTGATCTCATAACTTATTGATAAGGAAGAACATTATCTTTTGGCATACACCTTGCTGCAGCTCCCCGCA
GAGTTCCTGTGTCCCGGAGGTGCAGC**ATG**ACTACGCCACGCAAAGGCCCCCTTCTGTCATCACTCGTTCTGGCCCTGCTGGGTTGGG
AATCGACCAGCGAGGCGGCCGTGCAGTGCCAGCGCAC

mcoA (2665) and promoter area (P2665)

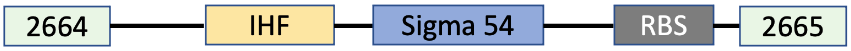

GGTAGTTGACCATGATCGGGTCCGGCAGGCTGCCGTCGCACAATTGGGTCACGAAACGGTGGCCAACGGCTGCCGACCAGGTCGGCT
CGTTCTGGCGGTGCAGGGACTGGCTGAAACGTTCGGT**CAT**GTTGCTGTCCTTGATGAGGGTGACAGCGAGACCTTGGCAGTTGGCAT
GGACTGGGCCTCGCAGTGAGGCCGGCAAAAAGGCAGGTTACAAGCCCCATCCCTTCGCCGGCATGATCCGGATCAGGTTCGAAGGGT
CACCGCGCTGCAGTGTTCAGCGGTTTCTCAGCCCATTGCCGGGCTCCCCTTGGTGGCGAACAGGTATACCCCAGTGCGTCGGGGCTG
TCACGCCCCGATCCACCCAGTGAGTGGGCATTGGGTCACCCGCTTTTGGGGACTTAGGCCCATTCTTGACGCGCCATTATTTAGCGC
ACCCTAGGCGCCGCAAGGTTTTGTATGACAAAGCATTACTGAAATACCTGTTGGCACAGCCGTTGCTCAGGCCCAAAGACCAGGCAA
AACGCCATTGATGGCCCTCAGCCATACAGCCGTGAGCGAGGCATAAGGCA**ATG**AAAACACCTGCCGTGATTCACCCGAACCGGTATG
TATTCCAGCTCTCTTCCGTCACCGCGCTGATGCTCAGCCTT

Supplementary Fig. 1. Genetic sequences for *mnxG* and *mcoA*. The top panel shows the sequence starting from gene 2446, with IHF, sigma 54 transcription factor, RBS, and 2447 (*mnxG*). The bottom panel shows the sequence starting from gene 2664, with IHF, sigma 54 transcription factor, RBS, and 2665 (*mcoA*).

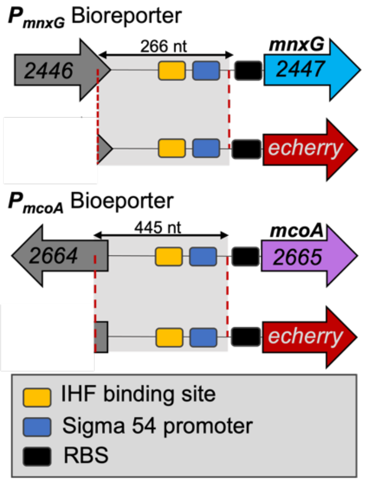

Supplementary Fig. 2. Schematic representation of the single bioreporter systems (P_{mnxG} and P_{mcoA}) used to monitor manganese oxidase gene promoter activity in *Pseudomonas putida* GB-1. Promoter regions (shown in grey and red dotted lines) devoid of RBS were fused to the *echerry* gene together with its RBS. Reporter fusions were inserted in a mini Tn5 delivery vector for subsequent single-copy integration into the genome of *P. putida* GB-1 containing the wild-type *mnxG* and *mcoA* loci. Genes are represented by arrow boxes. Each reporter was transformed into a separate strain. The scheme is not drawn to scale.

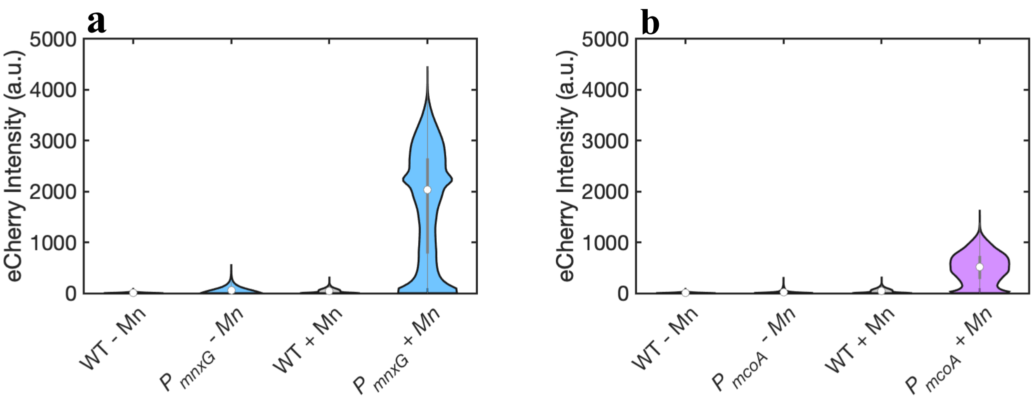

Supplementary Fig. 3. **Effect of Mn(II) on *P*$_{mnxG}$ and *P*$_{mcoA}$ activation in washed cultures**. Liquid cultures were washed at stationary phase and resuspended in MST salts with 50 µM $MnCl_2$ or without Mn. Samples were taken 24 h after washing for fluorescence measurements. (**a**) eCherry fluorescence signal distribution of wild type (gray) and bioreporter *P*$_{mnxG}$ (blue). (**b**). eCherry fluorescent signal distribution of wild type (gray) and *P*$_{mcoA}$ (purple). Data shown are for a single biological replicate for which more than 1000 cells were segmented.

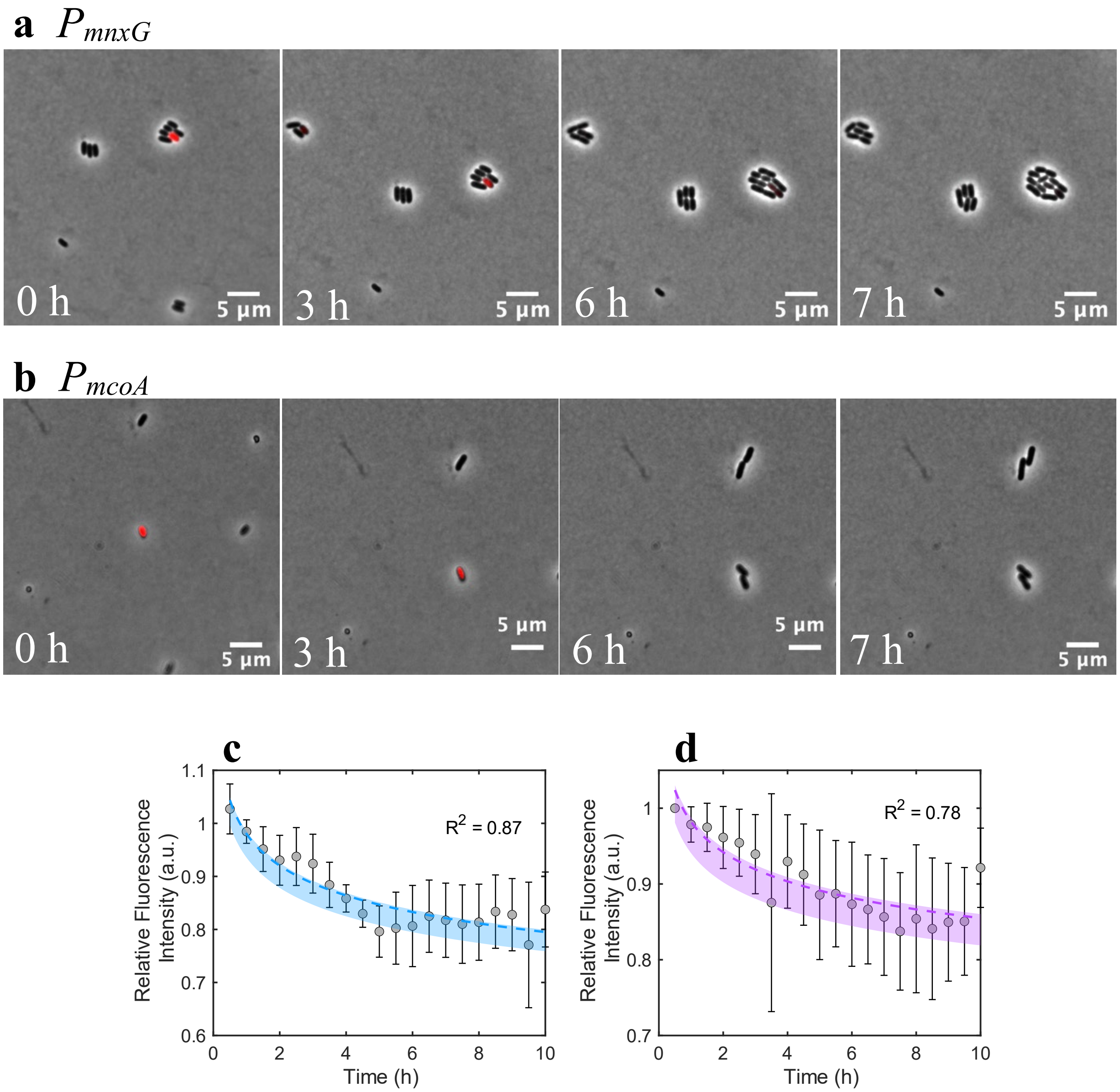

Supplementary Fig. 4. Re-growth of P_{mnxG} and P_{mcoA} reporting cells on MSTA agarose patches. Cells were first grown in liquid MSTA medium for 20h (for P_{mnxG}) and 48h (for P_{mcoA}) to ensure active bioreporter, and then transferred to a fresh MSTA agarose patch. Micrographs show the overlay of representative phase contrast and eCherry fluorescence images to illustrate the decrease in fluorescence intensity of P_{mnxG} (**a**) and P_{mcoA} (**b**) at 0 h, 3 h, 6 h, and 7 h. Reporting cells with active reporter P_{mnxG} and P_{mcoA} are shown in red. All images are rescaled to the same intensity. The relative average fluorescence intensity over time for three replicates of P_{mnxG} (**c**) and P_{mcoA} (**d**) are shown as grey symbols. The blue- and purple-colored areas show a decrease in average fluorescence signal with 95th confidence intervals, fitted as a power function. R^2 describes the power fitting relative to the average values. Results show a decrease in bioreporter activation after re-growth.

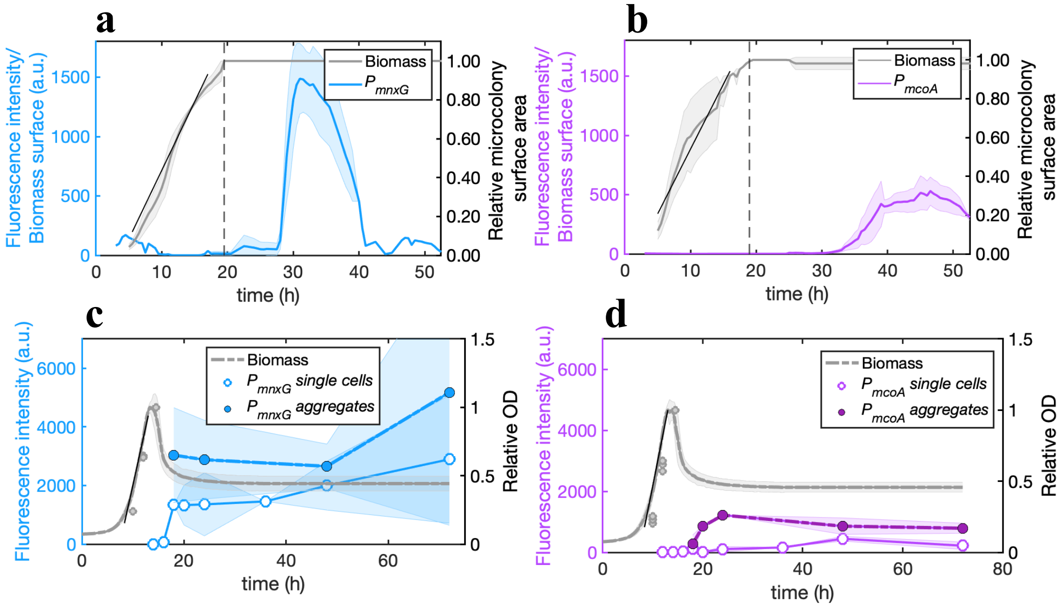

Supplementary Fig. 5. Temporal activation of the Mn oxidase promoters of *P. putida* GB-1 in surface-grown microcolonies and liquid-suspended growth. The sum of the fluorescence intensity normalized by microcolony size for (**a**) P_{mnxG} and (**b**) P_{mcoA} bioreporters, relative to the total surface area at entry into stationary phase at 19.5 h and 19 h, respectively (shown by the dotted vertical lines). The growth curve, calculated as the increase in microcolony surface, represents the average of 7 replicates for P_{mnxG} and 21 replicates for P_{mcoA}. The fluorescence intensity of P_{mnxG} (blue) is the average of 7 microcolonies, and P_{mcoA} (purple) is the average of 21 microcolonies. Shaded areas represent the 95th confidence interval. Growth rates (μ) were calculated during exponential phase. The sum of the fluorescence intensity normalized by biomass for (**c**) P_{mnxG} and **(d)** P_{mcoA}. The shaded area represents the standard deviation within triplicates. The plotted relative OD corresponds to the OD_{600} normalized by the maximum OD_{600}. Growth curves for liquid cultures were measured in 5 replicates on a plate reader. The gray dots represent samples obtained from liquid flasks and compared to the growth observed in 96-well plates to assess for differences in growth in 96-well plates and Erlenmeyer flasks.

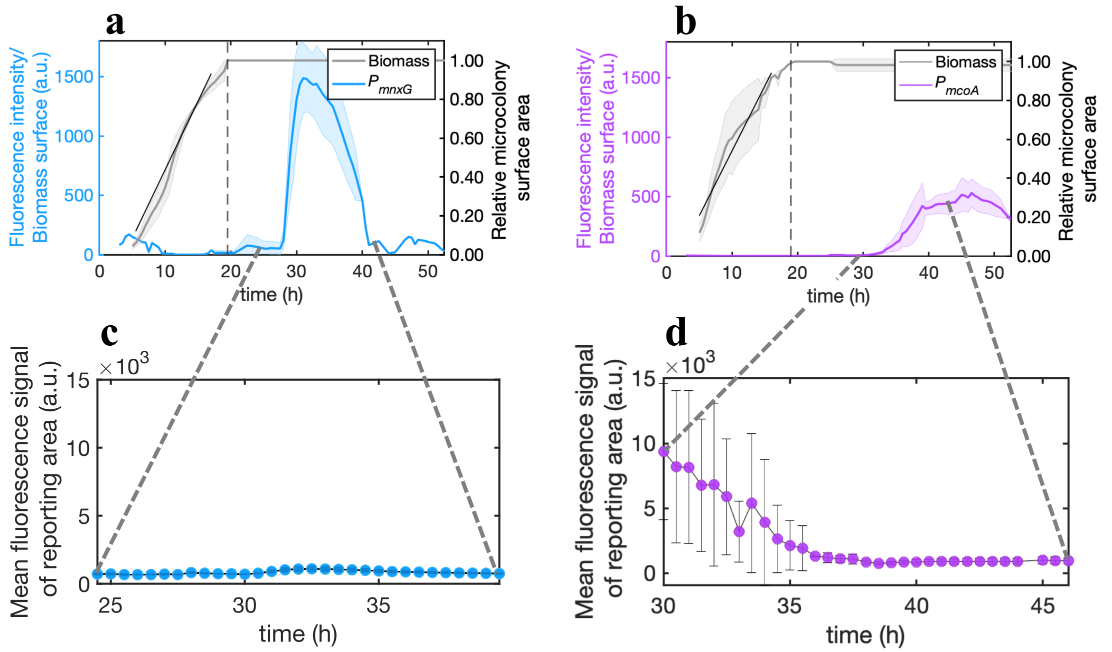

Supplementary Fig. 6. Mean fluorescence per reporting microcolony area over time. Overall reporter strain intensity P_{mnxG} (**a**) P_{mcoA} (**b**) relative to the total surface area at entry into stationary phase at 19.5 h and 19 h, respectively (shown by the dotted lines). Mean fluorescence signal per P_{mnxG} (**c**) and P_{mcoA} (**d**) reporting cell area. Results show no positive relationship between the average intensity with time, indicating that the overall increase in fluorescence intensity of P_{mnxG} and P_{mcoA}, observed in the **Main text, Figure 2**, is due to an increase in the proportion of the population activating the promoters P_{mnxG} and P_{mcoA}, and not due to an increase in fluorescence intensity per reporting cell. Note: the mean fluorescence signal of P_{mcoA} reporting cells from 30 h to 33 h comes from less than 0.7% of the population since most of the microcolony has inactive P_{mcoA}.

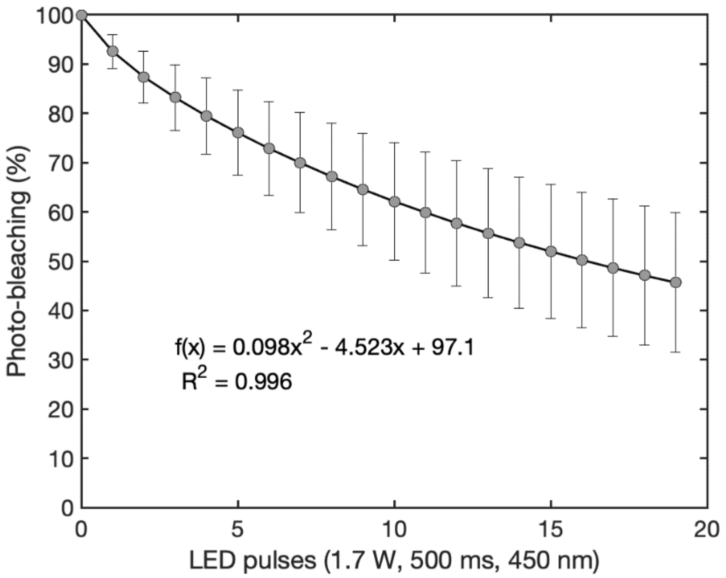

Supplementary Fig. 7. **Photobleaching due to prolonged LED exposure.** Photobleaching of 4 different sizes aggregates of P_{mnxG}. Aggregates were exposed to pulses of 500 ms LED in eCherry channel (emission at 450 nm) at 50% laser intensity (1.7W). The inset equation shows the second-degree fit for the average bleaching. The photobleaching rates measured on 72 h old microcolonies are calculated as 3.70 %/h, on average, and matched the decrease in P_{mnxG} intensity calculated from 31.5 h to 35.5 h (**Fig. 2a**). After this 4 h time window, the fluorescence intensity decreased rapidly at a rate of 16.34 %/h, until the fluorescence intensity was no longer detectable (**Fig. 2a**), showing inactivation of the promoter. The fluorescence from P_{mcoA}, after 47 h on solid surface, decreased at a rate of 6.78 %/h (**Fig. 2b**), which was greater than the experimentally determined photobleaching rate, suggesting that P_{mcoA} activation also ceased.

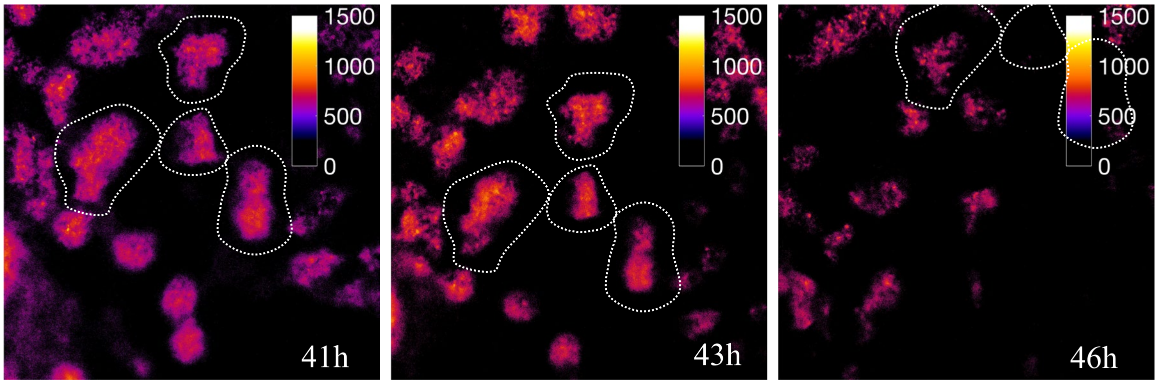

Supplementary Fig. 8. *P_{mcoA}* activation pattern from steady state to photobleaching. The proportion of the microcolonies with active ***P_{mcoA}*** does not increase further after reaching steady state (ca. 41 h) but decreases over time due to photobleaching. The shift in the location of the microcolonies within the frame is due to the drying of the agarose patch over time during extended time-lapses, shrinking it and shifting it from its set position.

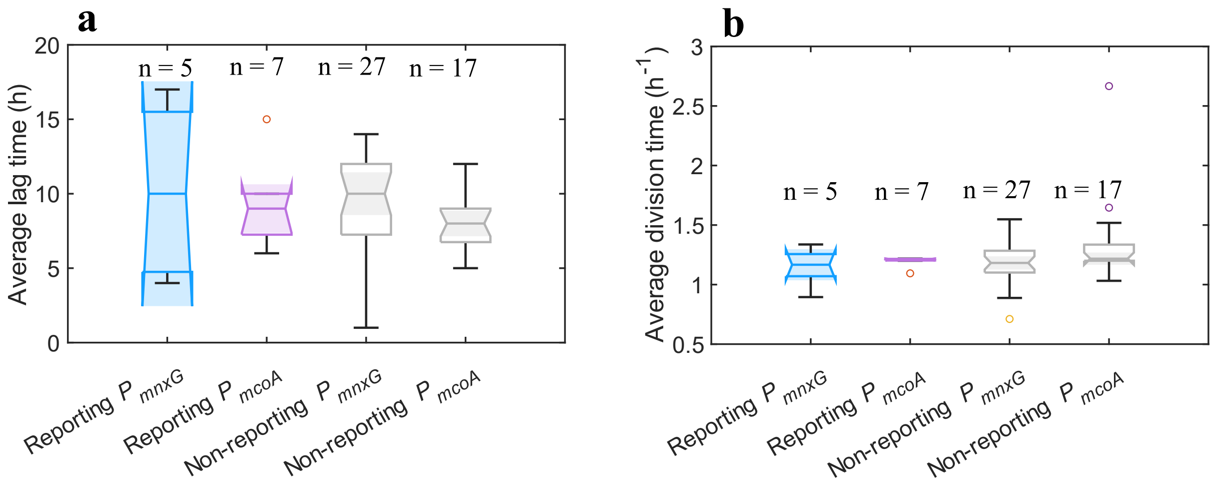

Supplementary Fig. 9. Viability of reporting and non-reporting cells. Cells were grown in MSTA containing 50 μM $MnCl_2$ for 48 h to generate a subpopulation of reporting cells. These cells were then washed and transferred to a 1% agarose patch containing 5 mM L-Arginine in MSTA. Cell division was monitored over 24h with a measurement made every 10 min and used to determine the average lag phase (**a**) and average division time (**b**) for reporting and non-reporting cells. ANOVA showed no significant differences between the reporting and non-reporting cells for both lag phase length (F>0.282, df=3) and division time (F>0.099, df=3). Note that the non-reporting cells may be expressing the untagged genes. However, the uniform distributions of the bioreporters suggest that the untagged genes did not skew the distribution.

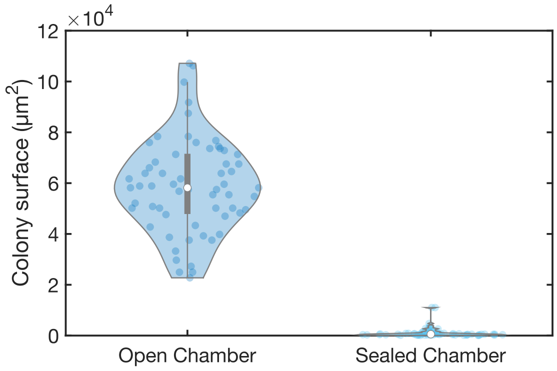

Supplementary Fig. 10. Surface area of the colonies cultured on MSTA solid surfaces (1% agarose) with 50 μM $MnCl_2$ in open and closed chambers. Data shows a greater than 100-fold increase in the mean surface areas of the microcolonies grown open to the air compared to the ones grown in closed chambers, which indicates that oxygen is the growth limiting factor in the closed microscope chambers.

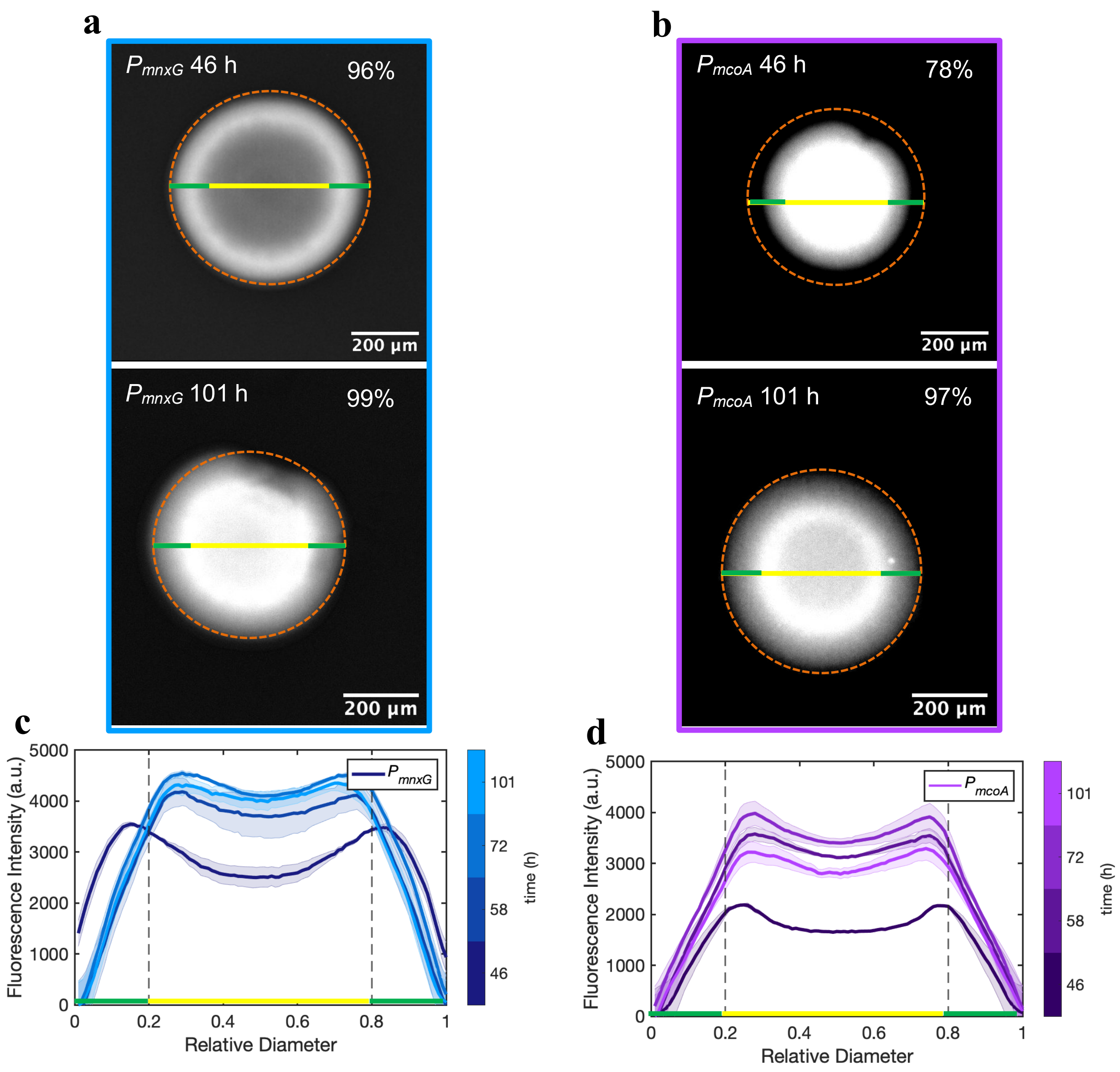

Supplementary Fig. 11. P_{mnxG} and P_{mcoA} activation patterns in open chambers colonies. Activation pattern of P_{mnxG} at 46 h and 101 h (**a**). Activation pattern **of P_{mcoA} at** 46 h and 101 h (**b**). The dotted orange lines represent the boundaries of the microcolony. The percentages represent the percent coverage of the microcolony with active bioreporter. Intensity profiles along the diameter of the microcolonies are shown in panel (**c**) for P_{mnxG} and panel (**d**) for P_{mcoA}. The profiles are the average of 5 replicates for each strain and each time point. The center of the profile is represented in red, and the outer rim is represented in green. Results show that P_{mnxG} activates first in the outer rim and then fills in. P_{mcoA} activates first in the center and then slowly progresses outwards. These patterns are consistent with the ones observed in the sealed-chambers microcolonies (**Main text, Fig. 3.**) and confirm that the fluorescence patterns are induced by the microcolony and not an artifact of the restrictive conditions found in sealed chambers.

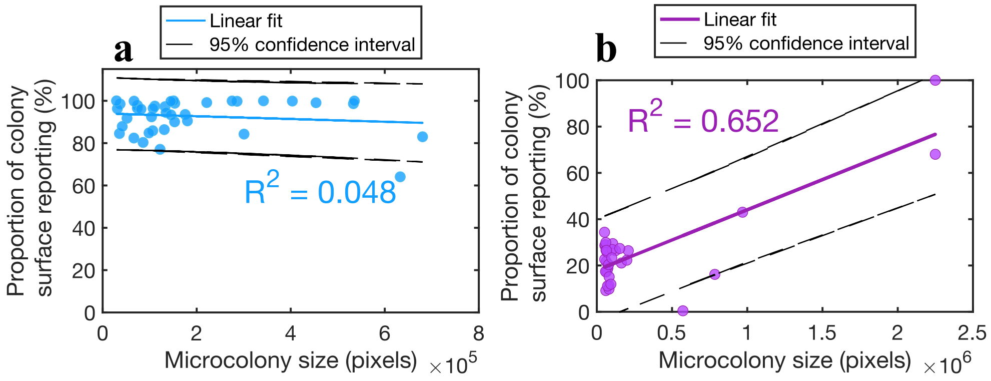

Supplementary Fig. 12. Relationship between colony size and proportion of the microcolony surface reporting. P_{mnxG} strain shows no linear relationship with colony size (n = 39) (**a**), whereas P_{mcoA} strain shows a linear relationship with colony size (n=33) with a p-value < 0.01 (**b**). Data originate from the 48 h time points in the time-lapse experiments presented in **Figs. 2** and **4** (Main text), with most of the P_{mcoA} size variability coming from the time-lapse in **Fig. 2**.

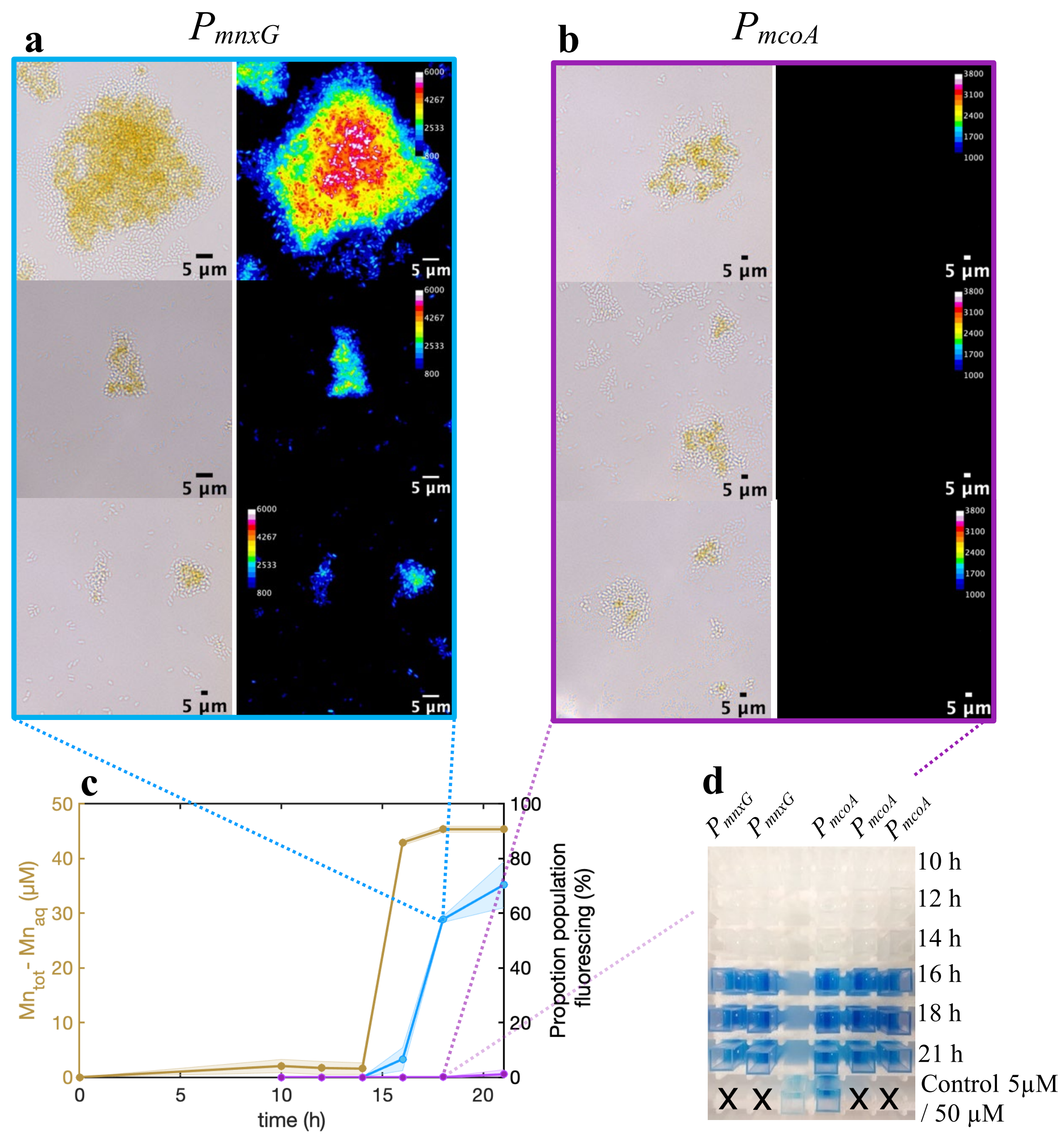

Supplementary Fig. 13. Mn(II) sorption and proportion of the population at mid-exponential phase and early stationary phase. (**a**) Representative brightfield color images of single cells and aggregates (P_{mnxG}), showing Mn oxide precipitates accumulating in the center of the aggregates (left column) and the corresponding P_{mnxG} reporter fluorescence (right column) at 18 h. (**b**) Representative brightfield color images of single cells and aggregates (P_{mcoA}), showing Mn oxide precipitates accumulating in the center of the aggregates (left column) and the corresponding P_{mcoA} reporter fluorescence (right column) at 18 h. (**c**) Proportion of population fluorescing over time for P_{mnxG} and P_{mcoA}. Patterns observed in **a** and **b**. indicate that the initial Mn oxide precipitation is attributed to P_{mnxG} and MnxG since no fluorescence was observed for PmcoA, but Mn oxides were present. The amount of Mn in the aqueous phase shows at most 3.9 ± 2.2% of sorption to the biomass prior to gene activation. (**d**) LBB colorimetric assay shows Mn oxide production starts at 16 h and coincides with P_{mnxG} activation. The LBB controls used δ-MnO_2 in MST salts at 5 µM and 50 µM concentrations. Data is shown in duplicates for P_{mnxG} and triplicates for P_{mcoA}.

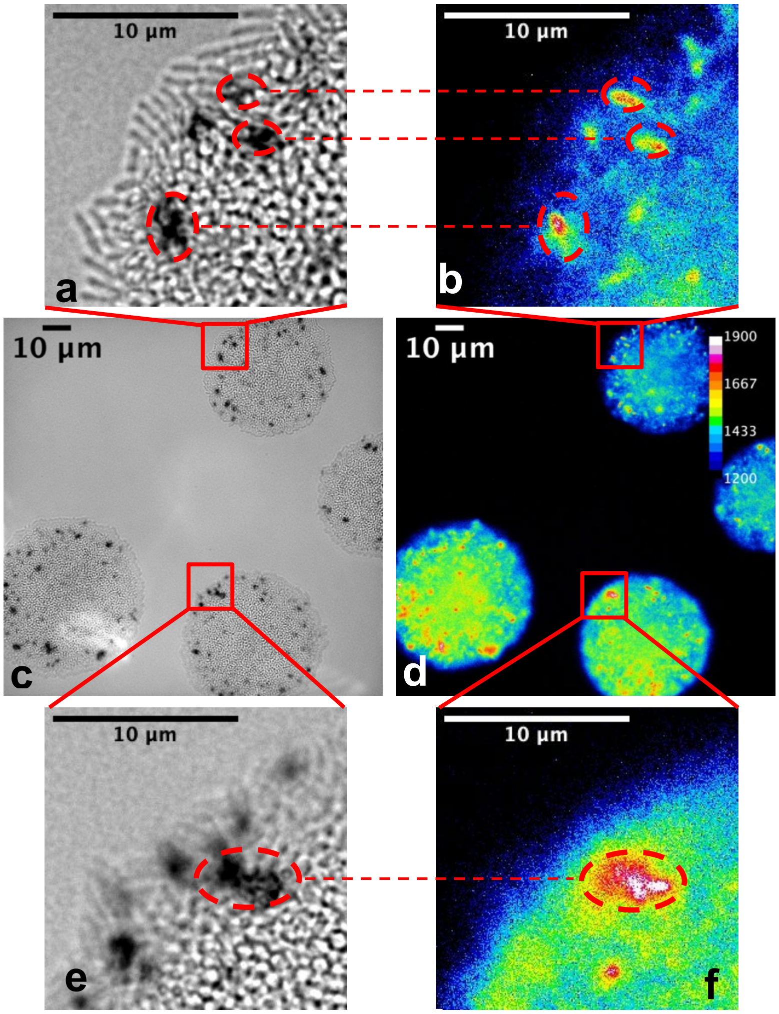

Supplementary Fig. 14. Micrographs showing co-location of hotspots of Mn oxide precipitates and promoter activation for the *P_{mnxG}* bioreporter. Brightfield images of microcolonies acquired at 40 h in the blue channel to delineate the Mn oxides (**c**) and heatmap of the fluorescence signal at 37 h (**d**). Panels **a**-**b** and **e**-**f** provide a close-up view of hotspots of Mn oxide (**a**, **e**) and P_{mnxG} fluorescence (**b**, **f**).

a

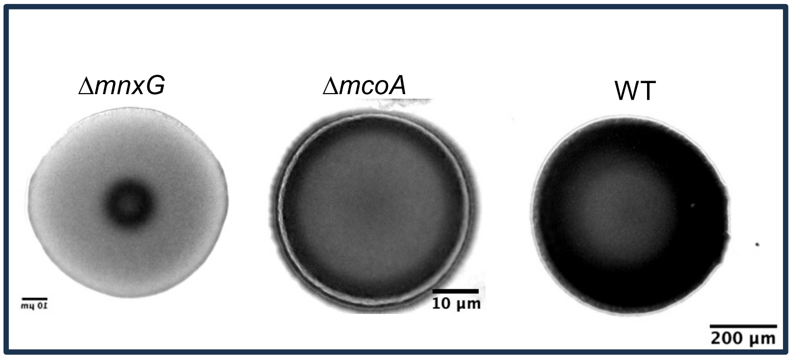

b

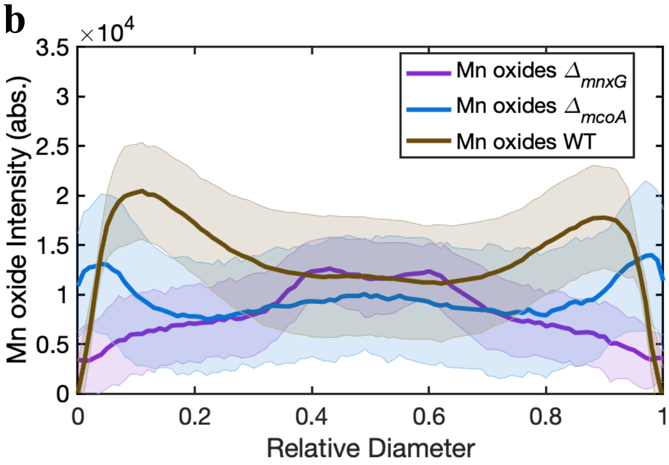

Supplementary Fig. 15. Mn oxide precipitation by the single knockouts and the wild type of *Pseudomonas putida* GB-1. The red subtracted blue channels or the Δ*mnxG,* Δ*mcoA*, and WT (**a**), highlighting the location of the Mn oxide precipitates. Average intensity profiles of the inverted color images for the single knockouts and WT (**b**). Averages of triplicates and the standard deviation are shown in a shaded area. Results show that McoA (Δ*mnxG*) produces Mn oxides only in the center, whereas MnxG (Δ*mcoA*) precipitates the oxides in the outer rim. The wild type shows Mn oxide precipitates in the whole colony, suggesting that each Mn oxidase is specific for local chemical conditions. The images were acquired at steady state (101 h). Note: the lighter shade in the center for the WT colony is an artifact of the image processing, resulting from darker biomass and thick layer of Mn oxides in the center that also shows a signal in the blue channel.

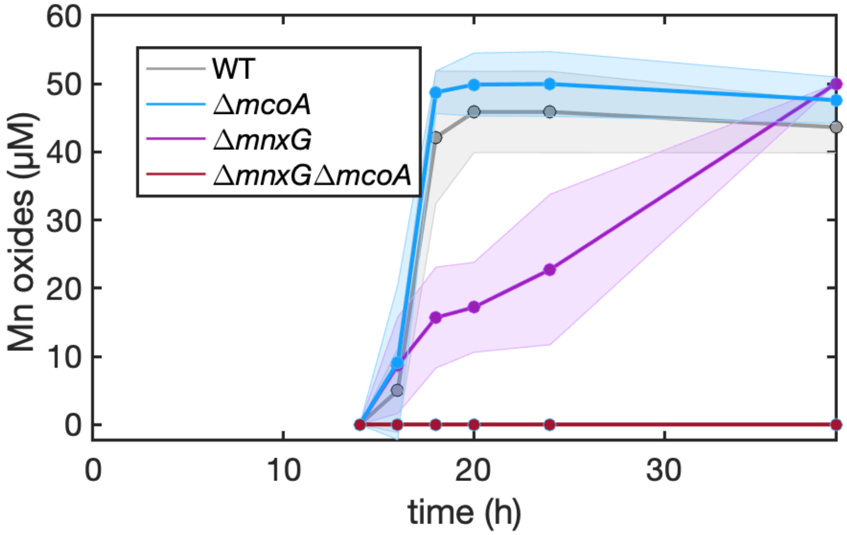

Supplementary Fig. 16. Mn oxidation kinetics of the wild type and single and double gene knockout strains. Data show that the Mn oxidation kinetics of the wild-type strain matches the Mn oxidation kinetics for the strain containing MnxG alone. While the strain containing McoA can eventually remove the 50 μM Mn(II) from the solution, the kinetics are much slower. The Mn oxide precipitated (y-axis) was calculated as the difference between the acid-digested Mn(II) and the aqueous Mn(II), and measured by ICP-MS. Shaded areas show the standard deviation within 3 replicates.

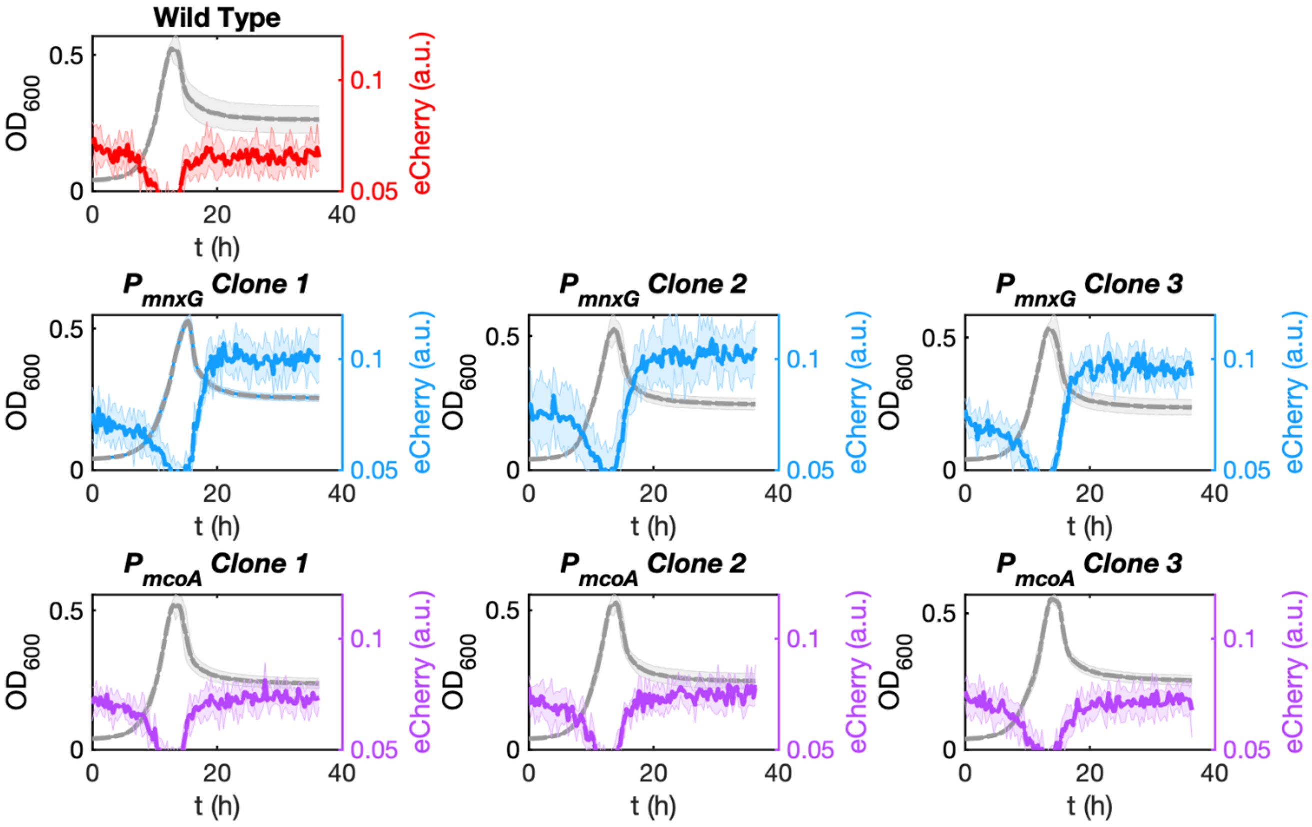

Supplementary Fig. 17. Growth and fluorescence of the-wild type (WT) and the bioreporter clones. Growth curves and fluorescence measurements were obtained using Varioskan LUX Multimode Microplate Reader at 30°C and under constant orbital shaking (160 rpm). Six replicates were measured for each strain in MSTA supplemented with 50 µM $MnCl_2$. Stationary phase was reached at 13h for all strains and replicates apart from the P_{mnxG} clone 1, reached stationary phase at 15h.

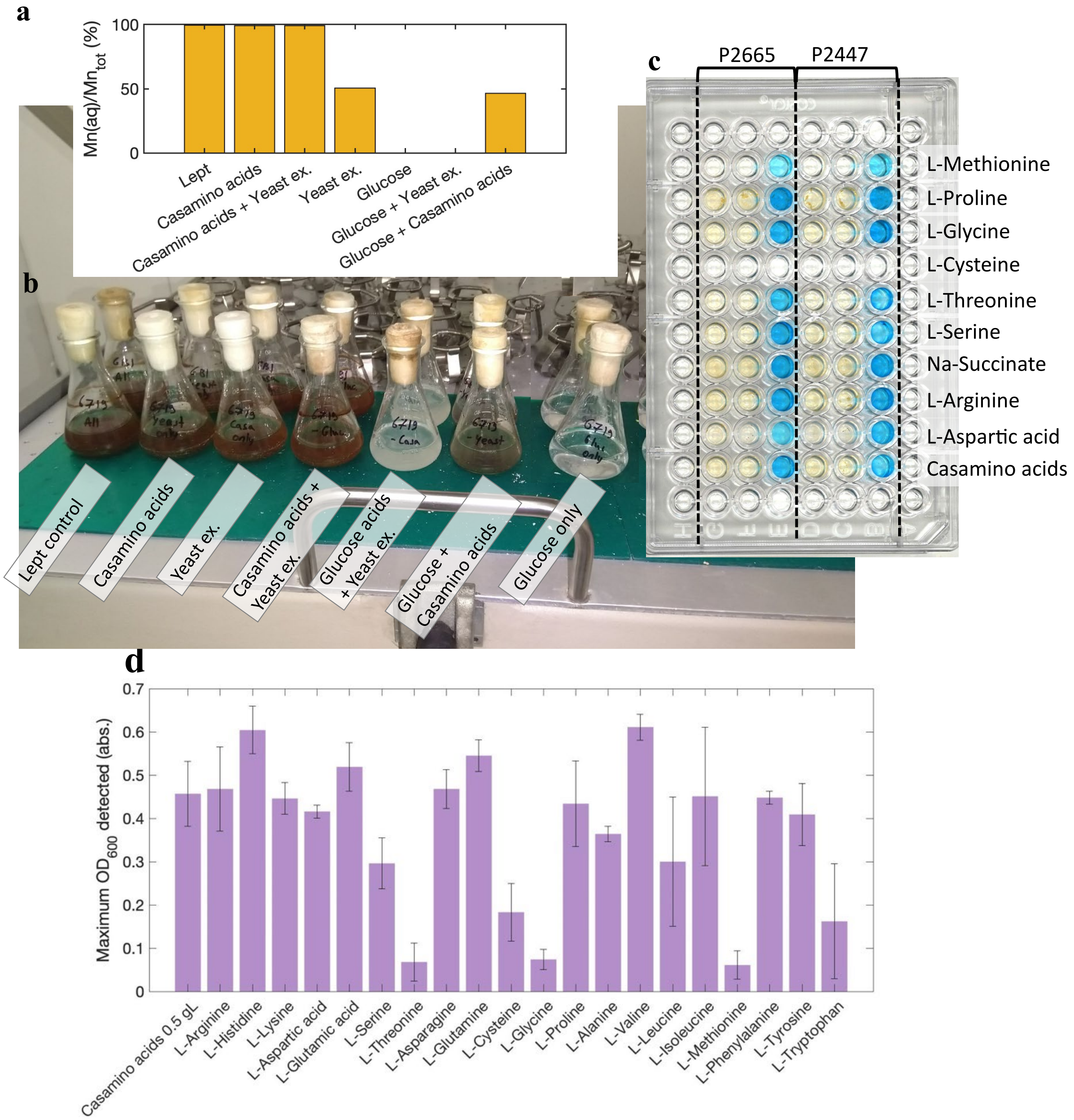

Supplementary Fig. 18. Development of MSTA growth medium. (**a**) Quantification of Mn oxides produced in Leptothrix salt supplemented with either Casamino acids, Glucose, or Yeast extract, or a mix. The percent of Mn oxides was calculated by the difference between total Mn and aqueous Mn, normalized to the total Mn. Quantification for a single replicate. (**b**) Visual brown Mn oxide precipitates in Leptothrix salts containing Casamino acids, Glucose, Yeast extracts, or a mix. Quantification in 6 replicates with LBB assay in duplicates. Results indicate that Casamino acids are the main inductors of Mn oxidation, whereas Glucose prevented Mn oxide precipitation. (**c**) Single amino acids (5 mM) were tested in MST salts and incubated in the plate reader at 30° and 180 rpm for 48 h, in triplicates per bioreporter strain. The amounts of Mn oxide precipitates were qualitatively assessed using LBB colorimetric assay. (**d**) Maximum growth was measured for the tested amino acids (each amino acid concentration was 5 mM). Results show that L-Arginine is the preferred carbon source with strong Mn oxide precipitation. All tested media were buffered at pH 7.0 using HEPES.

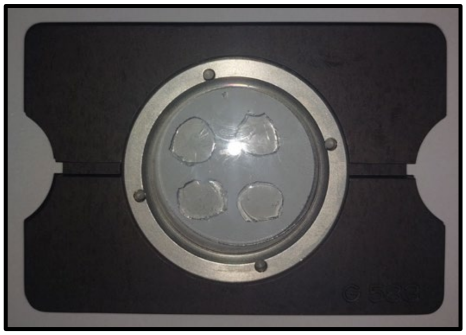

Supplementary Fig. 19. Microscopy chamber (Helmut Saur Laborbedarf, Germany), containing four agarose patches. The patches contained 1% agarose and MSTA growth medium with 50 µM $MnCl_2$. Two needles were introduced through open slits on the side of the chamber to improve oxygenation.

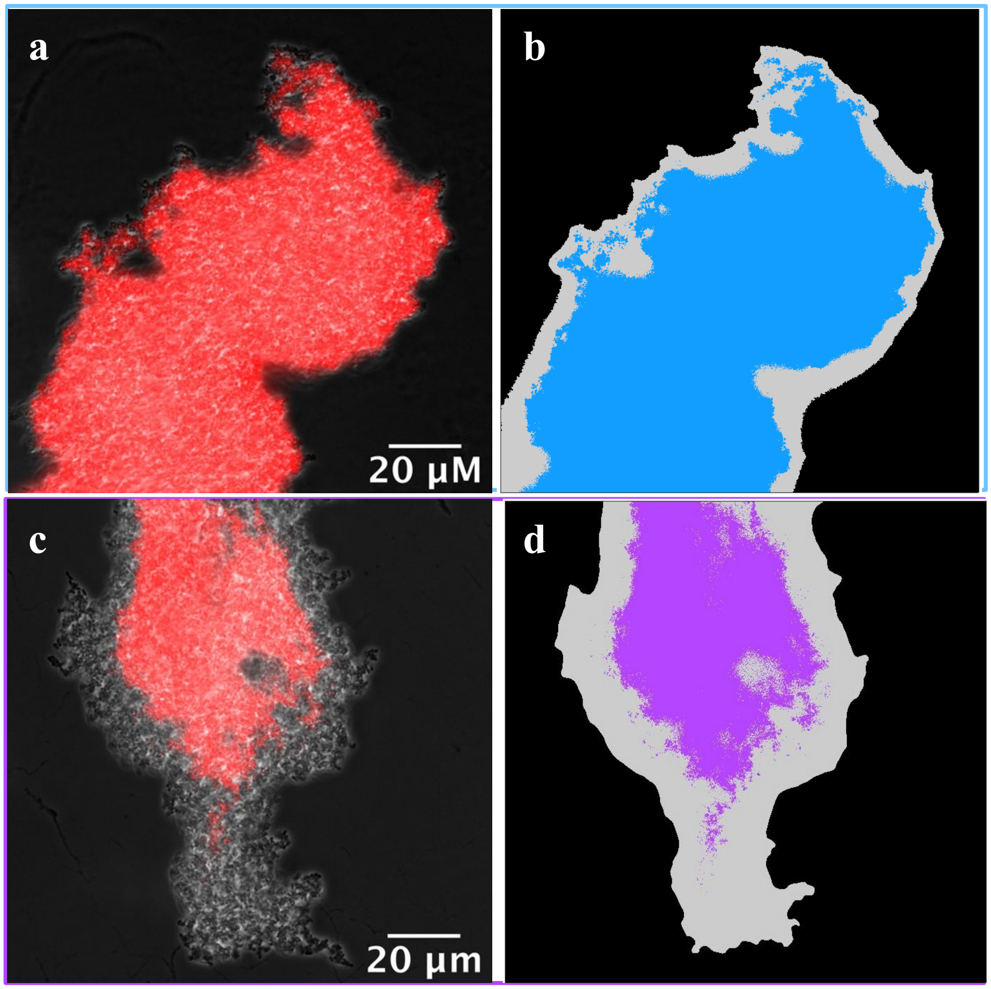

Supplementary Fig. 20. Analysis of the surface covered by reporting cells in aggregates. Representative image of phase contrast overlapped with eCherry fluorescence (in false red color, after applying the threshold) of the strain P_{mnxG} (**a**) grown in liquid with 50 μM $MnCl_2$ and the corresponding segmented mask (**b**). Representative image of phase contrast overlapped with eCherry fluorescence of the strain P_{mcoA} (**c**) grown in the same conditions and its corresponding segmented mask (**d**). The gray shows the aggregate area and the active reporters in color. The biomass segmentation is less accurate at the edges, with more biomass segmented than the reality. This results in a slight underestimation of the proportion of the biomass showing P_{mnxG} or P_{mcoA} activity.

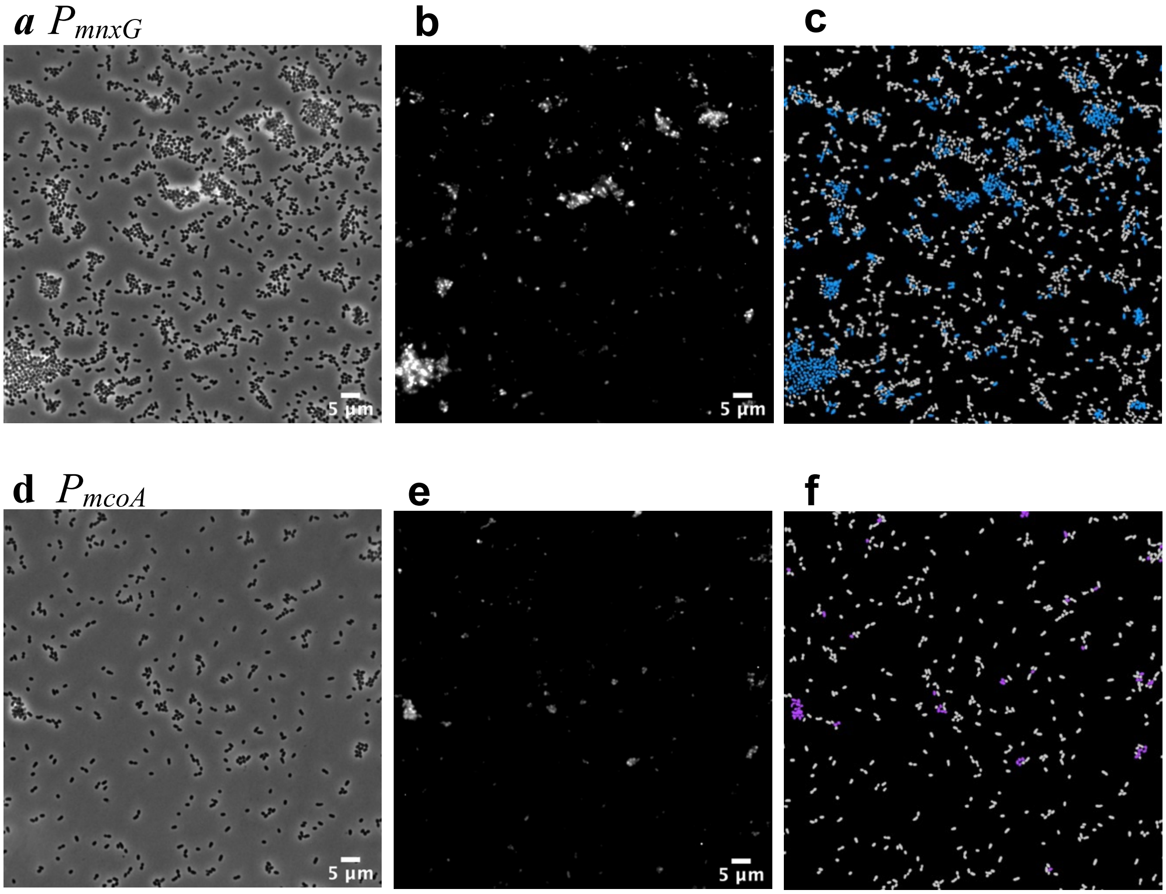

Supplementary Fig. 21. Representative image segmentation of single cells. (**a**) P_{mnxG} phase contrast image. (**b**) P_{mnxG} eCherry fluorescence image. (**c**) P_{mnxG} false-colored image output of the segmentation and identification of fluorescing cells, with 28.2% of the population fluorescing (in blue). (**d**) P_{mcoA} phase contrast image. (**e**) P_{mcoA} eCherry fluorescence image. (**f**) P_{mcoA} false-colored image output of the segmentation and identification of fluorescing cells, with 10.5% of the population fluorescing (in purple). All images acquired at 1000x, in the MSTA containing 50 μM Mn(**II**).

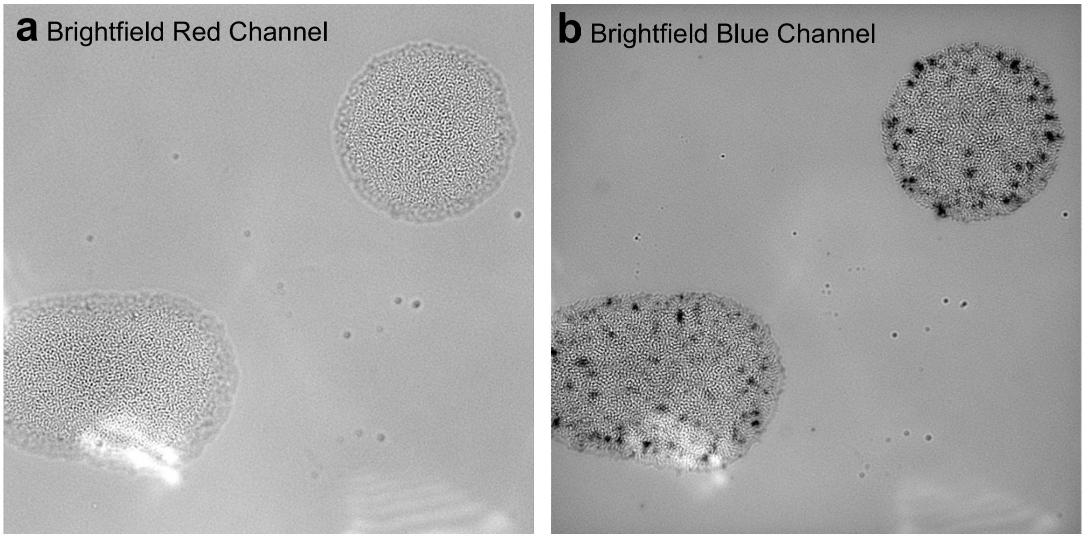

Supplementary Fig. 22. Color channel comparison for Mn oxide quantification. (**a**) Brightfield image acquired in the red channel. (**b**) Brightfield image acquired in the blue channel. The dark shadows indicate the location of the Mn oxide precipitates. Both channels are from the same original RGB color image.

Supplementary Table 1. Strains used in this study.

Strain Label	Description	Use
WT	*Pseudomonas putida* GB-1 Wild-type	Autofluorescence control
P_{mnxG}	*P. putida* GB-1::P2447_eCherry	Follow activation of promoter P_{mnxG} (*mnxG*)
P_{mcoA}	*P. putida* GB-1::P2665_eCherry	Follow activation of promoter P_{mcoA} (*mcoA*)
ΔmnxG	*P. putida* GB-1 single knockout of *mnxG*	Follow Mn oxidation by McoA
ΔmcoA	*P. putida* GB-1 single knockout of *mcoA*	Follow Mn oxidation by MnxG
ΔmnxGΔ mcoA	*P. putida* GB-1 double knockout of *mnxG* and *mcoA*	Mutant without the capacity to oxidize Mn
E. coli DH5α	Host containing pBAM1 plasmid used in the construction of PmnxG_eCherry or PmcoA_eCherry cassettes.	Construction of the fusion reporter strains

Supplementary Table 2. Primer used to construct the bioreporters and the knockout strains

Bioreporter strains	InFusion reaction to insert the promoter and *eCherry* gene into the pBAM1 vector digest with SmaI
PputGB1_2447.F	TTCGAGGCATGCCTGCAGCCCGGCCACTTTCCATACTGGACT
PputGB1_2447.R	ACCATGGCAGGTGCTCCTTCT CCGGGACACAGGAACTCT
PputGB1_2665L.F	TTCGAGGCATGCCTGCAGCCC CAGGCTGCCGTCGCACAAT
PputGB1_2665.R	ACCATGGCAGGTGCTCCTTCT GCTCACGGCTGTATGGCTGA
eCherry.F	agaa**ggag**cacctgccatggt
eCherryTn5.R	AATCAGAATTCGAGCTCGCCC ttatttgtacagctcatccatgcca

Deletions (Knockouts)	Cloning of upstream and downstream homologous fragment into pJP5603-ISceI
D2447UP.for	AGGGATAACAGGGTAATCTGAATTCGCGCTTGACCCCACCCCTGAA[2]
D2447UP.rev	TTTCCGTCACTGCGCCGGGGC GCGTGGCGTAGTCATGCTGCA
D2447DW.for	GCCCCGGCGCAG**TGA**CGGAAA
D2447DW.rev	GAAGCTTGCATGCCTGCAGGTCGAC GACCGTGTGGCGCAGCTGAT
D2665UP.for	AGGGATAACAGGGTAATCTGAATTC TCGAAGAAGGCCAGCTCCAA[2]
D2665UP.rev	ACGCGTCGGTTACTTGATGCTGAT GGCAGGTGTTTTCATTGCCTT
D2665DW.for	ATCAGCATCAAG**TAA**CCGACGCGT
D2665DW.rev	GAAGCTTGCATGCCTGCAGGTCGACCGGAAAATACGACAGTAAGAT

Supplementary Table 3. Defined growth medium composition of Mineral Salts and Traces L-Arginine (MSTA). All solutions were prepared with Milli-Q ultrapure water (18.2 MΩ·cm), and filter-sterilized with a 0.2 µm PES filter membrane. All reagents were stored at 4°C and prepared with ACS-grade chemicals. Final pH 7.0.

**Major Elements**	**Concentration in the growth medium**
$CaCl_2 \cdot 2H_2O$	0.4 mM
$MgSO_4 \cdot H_2O$	0.25 mM
Na_2HPO_4	0.25 mM
KH_2PO_4	0.15 mM
1:2 Fe(III)-EDTA	prepared by reacting 20 µM $FeCl_3 \cdot 6H_2O$ with 40 µM EDTA, adjusted at pH 6.5 with NaOH
HEPES buffer	10 mM, prepared by adjusting the pH to 7.0 with NaOH
$(NH_4)_2SO_4$	5 mM
**Trace Elements**	
$CuSO_4 \cdot 5H_2O$	40 nM
$ZnSO_4 \cdot 7H_2O$	273 nM
$CoCl_2 \cdot 6H_2O$	84 nM
$NaMoO_4 \cdot 2H_2O$	53.7 nM
**Carbon Source**	
L-Arginine*	5 mM

*L-Arginine was omitted for the preparation of the MSTA salts solution.

Supplementary Table 4. Characteristics of the bioreporter strains in MSTA (*SI Material and Methods*) containing 50 µM $MnCl_2$. Growth rates (calculated from the optical density at the exponential phase) and fluorescence were recorded using a Varioskan LUX Multimode Microplate Reader in 96-well plates. Mn oxide precipitation rates, onsets, and time to full oxidation were measured in batch experiments (Erlenmeyer flasks) as described in the method section.

	Growth rates (h^{-1})	Fluorescence intensity (a.u.)	Mn oxide precipitation rate (µM/h)	Mn oxidation onset (h)	Time to full oxidation(h)
WT	0.33	-	13.6 ± 5.7	16 ± 0.0	20.5 ± 2.2
P_{mnxG} - Clone 1	0.26	2.45	13.2 ± 8.1	16.5 ± 1.7	20 ± 2.8
P_{mnxG} - Clone 2	0.33	2.31	11.0	12	18
P_{mnxG} - Clone 3	0.33	2.39	22.6	16	18
P_{mcoA} - Clone 1	0.32	2.09	12.6 ± 5.5	16 ± 1.4	21 ± 3.0
P_{mcoA} - Clone 2	0.31	1.87	12.9	14	18
P_{mcoA} - Clone 3	0.28	1.78	23.9	16	18
